# Free will beliefs are better predicted by dualism than determinism beliefs across different cultures

**DOI:** 10.1371/journal.pone.0221617

**Published:** 2019-09-11

**Authors:** David Wisniewski, Robert Deutschländer, John-Dylan Haynes

**Affiliations:** 1 Bernstein Center for Computational Neuroscience, Charité-Universitätsmedizin Berlin, Berlin, Germany; 2 Department of Experimental Psychology, Ghent University, Ghent, Belgium; 3 Berlin Center for Advanced Neuroimaging, Charité-Universitätsmedizin Berlin, Berlin, Germany; 4 Clinic for Neurology, Charité-Universitätsmedizin Berlin, Berlin, Germany; 5 Berlin Institute of Health, Max Delbrück Center and Charité-Universitätsmedizin Berlin, Berlin, Germany; 6 Berlin School of Mind and Brain, Humboldt-Universität zu Berlin, Berlin, Germany; 7 Department of Psychology, Humboldt-Universität zu Berlin, Berlin, Germany; 8 SFB 940 Volition and Cognitive Control, Technische Universität Dresden, Dresden, Germany; Coventry University, UNITED KINGDOM

## Abstract

Most people believe in free will. Whether this belief is warranted or not, free will beliefs (FWB) are foundational for many legal systems and reducing FWB has effects on behavior from the motor to the social level. This raises the important question as to which specific FWB people hold. There are many different ways to conceptualize free will, and some might see physical determinism as a threat that might reduce FWB, while others might not. Here, we investigate lay FWB in a large, representative, replicated online survey study in the US and Singapore (n = 1800), assessing differences in FWB with unprecedented depth within and between cultures. Specifically, we assess the relation of FWB, as measured using the Free Will Inventory, to determinism, dualism and related concepts like libertarianism and compatibilism. We find that libertarian, compatibilist, and dualist, intuitions were related to FWB, but that these intuitions were often logically inconsistent. Importantly, direct comparisons suggest that dualism was more predictive of FWB than other intuitions. Thus, believing in free will goes hand-in-hand with a belief in a non-physical mind. Highlighting the importance of dualism for FWB impacts academic debates on free will, which currently largely focus on its relation to determinism. Our findings also shed light on how recent (neuro)scientific findings might impact FWB. Demonstrating physical determinism in the brain need not have a strong impact on FWB, due to a wide-spread belief in dualism.

## Introduction

For centuries scholars have been debating whether humans have free will or not. But independent of this academic, philosophical debate, most laypeople maintain a strong belief in free will [1–3]. Many claim that this is socially desirable because the less people believe in free will the more they are prone to aggression [4] and such beliefs are fundamental to much of our behavior [5]. Others highlighted possible pro-social effects of reduced FWB [6]. Importantly, free will beliefs (FWB) are intimately linked to our sense of responsibility and are a foundation for many criminal justice systems [7]. If FWB changed in the general public, this could potentially also change e.g. our sense of responsibility in un-intuitive ways. According to some, such changes might be triggered by recent neuroscientific findings [8,9]. Based on the idea of physical determinism, i.e. that all physical events are inevitably caused by prior physical events, such studies often assess whether or how behavior is caused by a prior neural event. Some take this as dis-proving free will [10,11], although this notion remains contested [12]. Despite this ongoing debate, neuroscientific findings are already being used as an argument for reduced responsibility in some criminal cases [7,13].

### Academic and lay theories of free will

It thus seems that FWB might change over time, with potential broader effects on behavior. However, in order to assess just how FWB might change, we first need to understand which specific FWB people hold in the first place. In the philosophical, academic debate, at least three broadly different theories have been put forward to support FWB. First, one might believe that free will is the ability to do otherwise even if two choice situations were identical. This notion is incompatible with physical determinism, which in turn is often rejected to make space for free will (libertarianism, which is a prominent incompatibilist position [14]). Second, one might believe that free will is the ability to act according to (or suppress) one’s drives and desires, and/or not being coerced by external forces. This notion is compatible with determinism (compatibilism, [3,12]). In this case, showing how our behavior is physically determined poses no threat to free will. Third, one might believe that the mind or soul is not a physical entity (dualism, [15]), which potentially also removes potential threats to free will. These positions have been clearly defined in the academic debate, and arguments for and against them are well known [12]. Here, we only present the core beliefs of each theory. In fact, there exist many versions of each of the three main positions, which are described in much more detail elsewhere [11,12,14]. Recently, there has also been growing interest in lay theories of free will and related intuitions in the general public [16,17], with much research revolving around the question whether laypeople are intuitive compatibilists or incompatibilists [18,19]. This work thus revolved around the relation of free will and determinism, and results have often been inconclusive as lay theories often are inconsistent [19]. Agreeing with e.g. libertarian stances on free will does not necessarily mean one would act in accordance with this view, and one goal of this study was to measure both agreement to explicit theoretical statements as well as behavior following from these stances (for some initial evidence see [1]).

One factor that received relatively less attention in the past is the relation of free will to dualism. One recent empirical investigation assessed the relationship of different forms of dualism and free will beliefs [20]. The authors found substance dualism (i.e. the mental and the physical are separate, independent substances) to be more strongly related to free will beliefs than reductive physicalism (i.e. there is only physical matter, making all mental states physical), both of which are different views on mind-body dualism. They did not however differentiate between different views on the relation of free will and determinism (e.g. compatibilism, libertarianism). Here, we aim to compare the link of FWB to both determinism and dualism simultaneously, assessing which theory best explains lay beliefs. Additionally, previous work on this issue was performed mostly on US undergraduate samples (e.g. [21,22]), and it thus remains unclear whether results are truly representative of lay beliefs in the general public. Investigating lay theories in more representative samples has received little attention in the past. If lay beliefs are to inform academic debates on free will, which has been argued for in the past [15,23], these lay beliefs need to be investigated in large, representative samples however. In sum, while the academic debate largely revolves around reconciling the seemingly contradictory notions of free will and determinism, whether determinism indeed challenges free will in the eyes of the general public remains unclear. Here, our main goal was to determine whether FWB are best explained by compatibilist, libertarian, or dualist intuitions in the general public.

### The present investigation

Here, we present data addressing this issue directly. In an online survey study, we acquired data from n = 900 subjects in the United States (US), and n = 900 subjects in Singapore (SGP), drawn representatively for sex and age. It has been suggested before that many findings in psychological research lack generalizability because of selective and biased sampling procedures [24], which is especially true for empirical research on free will [25,26]. In contrast to most previous research, our samples are representative for each of the two cultures tested, and we included one western culture, which is regarded as individualist, and one east Asian culture, typically taken to be collectivist [27]. Previous research demonstrated that locus of control is less external in individualistic countries, as compared to collectivistic countries [28,29], and FWB are in turn correlated with locus of control ([2], and also S1 Analysis). We thus initially hypothesized that FWB might be stronger in the US than in SGP. Additionally, we might expect a higher prevalence of libertarian views in the US, as generally individualistic participants might be more strongly opposed to their behavior being determined by external forces (i.e. physical determinism). FWB were measured using the Free Will Inventory [1], which separately measures people’s free will beliefs along three dimensions: general free will (FW-general, “Do we have free will or not?”), determinism (FW-de, “Is the world determined by prior events or not?”), and dualism (FW-du, “Is the mind a non-physical entity or not?”). We then assessed the relationship of general free will beliefs to determinism beliefs, hypothesizing that they are related to determinism beliefs based on prominent positions in the philosophical discourse (e.g. compatibilism). We also assessed the relationship of general free will beliefs to dualism, an issue that received increased attention recently [20]. We had no strong a priori hypotheses regarding the relation of free will and dualism. To our knowledge this is the largest in-depth analysis of FWB to date (see [27] for preliminary evidence).

## Materials and methods

### Participants

We acquired data from 900 subjects in the US (age between 18 and 64, mean age = 41.1, 459 females, 441 males, see Table 1 for full sample characteristics, n(intended) = 900, n(achieved) = 900). We further acquired 900 subjects in Singapore (SGP, age between 18 and 64, mean age 38.9, 450 females, 450 males, n(intended) = 900, n(achieved) = 900). Both samples are representative with respect to sex, which was the main reason for using this large sample size. Age was binned into five different age groups (18–24, 25–34, 35–44, 45–54, 55–64), and the US sample is fully representative with respect to age. In the SGP sample, we were unable to recruit the necessary number of participants in the oldest age group (55–64, see Table 1), and the age distribution is thus slightly skewed towards younger subjects. This deviation is very small however, and should not strongly impact the representativeness of our SGP sample. All subjects were contacted by and recruited through a professional polling institute during the summer of 2016 (ResearchNow, now known as Dynata, www.dynata.com), and were reimbursed for their participation. The polling institute is licensed to operate in the US and SGP, and followed all applicable local regulations. All participants first opted in to be included in a list of potential participants for ResearchNow, and then again gave their written informed consent to participate in this specific study. ResearchNow was unaware of the purpose of the current investigation, and ensured all participants remained anonymous. No personally identifiable data was ever given to the researchers. This study was approved by the ethical committee of the Department of Psychology, Humboldt University, Berlin, and was conducted in accordance with the Helsinki declaration.

**Table 1:** Sample characteristics in the USA and Singapore.

	US			SGP		
	Target	Empirical data		Target	Empirical data	
**Age**	RF	RF	N	RF	RF	N
18–24	13%	13%	117	15%	15.5%	140
25–34	22%	22%	198	22%	22.5%	203
35–44	22%	22%	198	25%	25.6%	231
45–54	24%	24%	216	24%	24.7%	223
55–64	19%	19%	171	15%	11.4%	103
**Sex**						
male	49%	49%	441	50%	50%	450
female	51%	51%	459	50%	50%	450
total	100%	900		100%	900	

Shown are the target age (by age group) and sex distributions (Target), as well as the actual distributions in both samples (Empirical data), RF = relative frequency, N = absolute number, US = United States, SGP = Singapore.

Please note that we decided to acquire data in two cultures in order to better test the generalizability of our results. For this purpose, the two cultures need to differ significantly along at least one dimension. Here, we focus on two countries that differ mainly in their individualism scores (US = highly individualistic, SGP = highly collectivistic, [30]). Unfortunately, we were unable to directly measure individualism in our samples due to time constraints. However, it seems highly unlikely that the US and SGP samples are equal in their individualism scores in our study, given our large samples and previous work demonstrating that these two countries lie at both extremes of the individualism spectrum [31]. Any potential cultural differences are likely to be driven at least in part by differences in individualism, but given the fact that these two cultures also differ along other dimensions, cultural differences should be interpreted with care. In order to address at least some of the potential alternative explanations, we controlled for differences in demographic variables (age, sex, education, see below).

### Measures

All measures were administered in their English version to all subjects, in order to maximize comparability between the different samples.

#### Free will beliefs

Free will beliefs were measured using the Free Will Inventory [1], which was developed to measure beliefs about free will and related concepts. The FWI (part 1) captures FWB along three dimensions: free will (FW-general), determinism (FW-de), and dualism (FW-du). Each sub-scale was measured using 5 items, with a total of 15 items. Responses were based on a 7-point Likert-type scale ranging from 1 (strongly disagree) to 7 (strongly agree), the mid-point of the scale was 4 (neither agree nor disagree). For illustration purposes, we rescaled all items to fall in between -1 (strongly disagree) and +1 (strongly agree). Thus, positive values indicate agreement, while negative values indicate disagreement (0 indicated neither agreement nor disagreement). The reliability of the subscales (as computed using the *psych* package in *R*) was acceptable in the USA and SGP (Table 2). In order to test whether the FWI gives a similar factor structure in both samples, and to replicate [1], we performed additional validation analyses (see S2 Analysis). We also administered the second part of the FWI, which measures subjects’ agreement to compatibilist views on free will, as well as their intuitions on punishment and responsibility. The FWI has no reverse-coded items, and is thus vulnerable to acquiescence bias. To rule out such biases, we performed an additional control analysis. Using data from a different questionnaire in the same sample (self-efficacy, see below), which contains reverse-coded items, we showed that subjects did not demonstrate acquiescence bias (see S3 Analysis).

**Table 2:** Reliability of the FWI.

	US		SGP	
	alpha	95% CI	alpha	95% CI
FWI				
free will	0.81	0.79–0.83	0.79	0.77–0.81
determinism	0.85	0.84–0.87	0.81	0.79–0.83
dualism	0.82	0.80–0.84	0.78	0.76–0.80

Cronbach’s alpha coefficient for all sub-scales reported. Includes 95% confidence intervals (CI). FWI = free will inventory, US = United States, SGP = Singapore.

#### Demographic variables

We recorded the following demographic variables: age, sex (male, female), highest level of education (pre-primary, primary, lower secondary, upper secondary, post-secondary non-tertiary, short-cycle tertiary, bachelors, masters, doctors, no answer), total years of education, and profession (open responses).

#### Other measures

We also acquired data from other measures, including locus of control (results reported in S1 Analysis), self-efficacy, neuroticism, self-esteem, current bodily state. The focus of this investigation is the description of FWB and their relation to determinism and dualism, and results from these additional measures are thus not reported here.

### Procedure

After being invited to participate, subjects received a link to the online questionnaires. Subjects completed all questionnaires online in the browser, as implemented in the Unipark software (QuestBack GmbH, Köln, Germany). First, they gave their written informed consent to participate in the study. Instructions were then presented on screen, explaining how to indicate responses to each item. The study always started with acquiring demographic data. After this part was completed, subjects were presented with the questionnaires, in a randomized order to avoid sequence effects. Subjects were given no time constraints for responding to the items.

### Analyses

All analyses were performed in *R* (RStudio, Version 1.1.456, RRID:SCR_000432).

#### Replication procedure

Replications remain a rarity in psychological research on free will [26,32], but crucially, all statistical inferences reported here were replicated within two independent samples. We first randomly split the US sample in half (n = 450 for each sub-sample), with the constraint that both sub-samples remain representative of sex and age, just as the original sample was. The first half was called *main sample*, the second *replication sample*. The same procedure was performed in the SGP sample. All inference statistics were performed on both independent samples. In this report, we only present results that were confirmed in both the main and in the independent replication sample.

#### Descriptive statistics

In order to first describe the results in both cultures, we counted how many subjects believed, disbelieved, or were indifferent towards free will (FW-gen), determinism (FW-de), and dualism (FW-du). For this purpose, we averaged item responses separately for each of the three FWI sub-scales, and then assessed the proportion of subjects scoring positively (belief), negatively (disbelief), or zero (indifference). This was done separately for both cultures and all 3 sub-scales. Given the wording of the items, and especially the rating scale labels used (strongly agree, neither agree not disagree, strongly disagree), we believe responses can be interpreted meaningfully in the way described here.

#### Analysis 1: Libertarianism

In order to assess whether libertarianism provided a good explanation for free will beliefs [14], we performed three different analyses. First, part 2 of the FWI contains items that explicitly assess agreement to central tenets of libertarian, incompatibilist theories of free will. One item assesses the ‘ability to do otherwise’ (item 1: ‘Free will is the ability to make different choices even if everything leading up to one’s choice (e.g., the past, the situation, and their desires, beliefs, etc.) were exactly the same.’), a central component of incompatibilist definitions of free will. Another item assesses the notion of the unmoved mover (item 6: ‘To have free will is to be able to cause things to happen in the world without at the same time being caused to make those things happen.’). Many libertarians would agree to both claims, and we thus assessed how many participants agreed to these items separately in the US, and SGP. Responses to these items by themselves are agnostic as to whether participants actually believe in free will and/or determinism or not. They merely ask whether participants agree with key concepts and definitions of (some) libertarian, incompatibilist views. Results from these items thus need to be seen in context with the actual data from the general free will sub-scale. By e.g. showing a positive correlation with FW-gen, we can show that a strong belief in free will co-occurs with an acceptance of incompatibilist definitions of free will. This in turn would be more direct evidence for libertarian intuitions of free will than e.g. only showing agreement with the libertarian intuitions. In order to test the relationship of these two items and the FW-gen sub-scale, we used a linear model approach. We estimated two simple Bayesian linear models (using *lmBF* from the *BayesFactor* package), one for each item, entering FW-gen as the dependent, and responses to the above mentioned items as the independent variable (e.g. FW-gen ~ FWI part2 item1). Please note, that we refrained from combining both items into a ‘libertarianism’ index through e.g. averaging responses because items of FWI part2 showed insufficient psychometric properties to construct a e.g. ‘libertarianism’ sub-scale [1]. We thus analysed all FWI part2 items independently. We assessed whether the models explained FW-gen by computing the Bayes Factor (BF_10_), which indicates whether effects are present, absent, or whether the data is inconclusive [33]. We considered BF_10_s from 0.3 to 1 as anecdotal evidence for the absence of an effect. BF_10_s from 0.1 to 0.3 were considered moderate, while BF_10_s < 0.1 were considered strong evidence for the absence of an effect. BF_10_s from 1 to 3 were considered anecdotal, from 3 to 10 as moderate, and above 10 as strong evidence for the presence of an effect, respectively. Although frequentist hypothesis testing does not allow us to test evidence in favor of H_0_, we report their results for the interested reader (using *lm*). Our conclusions are solely based on the Bayesian models however.

Explicitly agreeing to an incompatibilist definition of free will, which underlies libertarian theories, does not automatically mean that one acts consistently with this belief. In a second analysis, we therefore assessed whether participants responded to part 1 of the FWI in a way that is consistent with libertarian positions. If a large portion of participants indeed agreed with an incompatibilist definition of free will, one might expect them to only believe in free will but not in determinism. In order to test this prediction, we assessed the relation of FW-gen and FW-de, separately for US and SGP, again using a similar Bayesian linear model approach (FW-gen ~ FW-de), and expected a negative relationship. The more subjects believed in free will, the less they should believe in determinism. Again, we base our conclusions on the estimated BF_10_, but report frequentist statistics for the interested reader as well.

Third, the relationship of FW-gen and FW-de can be assessed by comparing data across both countries. Libertarianism predicts that if we see cultural differences on the FW-gen scale (e.g. US > SGP, as we expected based on previous findings [2,28,29]), we should observe differences in the opposite direction on the FW-de scale (e.g. SGP > US). This was tested using Bayesian two-sample t-tests (using *ttestBF* from the *BayesFactor* package), which output BF_10_s. These were interpreted as described above. We again report frequentist tests for the interested reader, but base our conclusions on BF_10_s.

#### Analysis 2: Compatibilism

In order to assess whether compatibilist intuitions explain free will beliefs [12], we used a similar analysis approach as in Analysis 1. First, we assessed two items of part2 of the FWI that probe agreement to two central claims of compatibilist theories of free will. Item 2 assessed the notion that free will means choosing according to one’s beliefs and desires (‘Free will is the ability to make a choice based on one’s beliefs and desires such that, if one had different beliefs or desires, one’s choice would have been different as well.’). Item 7 assessed the notion that free will means not being coerced by other people (‘People have free will as long as they are able to do what they want without being coerced or constrained by other people.’). Again, we first counted the number of subjects agreeing to these statements. Given that these items are again agnostic as to whether subjects actually believe in free will, we again assessed whether responses to these items were related to FW-gen scores using a linear model approach (e.g. FW-gen ~ FWI part2 item2). If FW-gen is linked to compatibilist theories of free will, we expect to see a positive relationship. Similar to Analysis 1, we can make predictions about responses to part 1 of the FWI if a majority of participants agreed with compatibilist definitions of free will. In this case we would expect either no or a positive correlation between FW-gen and FW-de, as subjects can (but do not have to) believe in free will and determinism at the same time. This was tested using the same linear model approach as in Analysis 1 (FW-gen ~ FW-de). Please note that the FW-de sub-scale does not differentiate between different sources of dis/belief in determinism (e.g. fatalistic vs scientific determinism, [20]), but it still allowed us to distinguish between libertarian and compatibilistic theories of free will, a comparison that has not been made explicit in most previous research.

#### Analysis 3: Dualism

In order to assess whether free will beliefs are related to dualistic intuitions [15], we again followed the same analysis approach as before. We first assessed agreement to an item in part2 of the FWI, which assessed the necessity of an immaterial soul for free will (item 5: ‘If it turned out that people lacked non-physical (or immaterial) souls, then they would lack free will.’). We then computed linear models to assess whether responses to this item were positively related to FW-gen (FW-gen ~ FWI part2 item5). If a majority of the subjects believed an immaterial soul to be a necessary condition for free will, we would further expect a positive relationship between FW-gen and FW-du, which we tested using a linear model approach again (FW-gen ~ FW-du). The FW-du subscale covers two aspects of dualism beliefs, substance dualism and non-reductivism, and both potentially contribute to observed results. As before, all these analyses were performed separately for the US and SGP.

#### Analysis 4: Comparing different intuitions

Lay theories on philosophical issues rarely are logically consistent [34], which is one reason why we assessed both the explicit agreement to central claims of libertarian, compatibilist, and dualist theories, and whether behavior was consistent with their predictions. However, it is likely that more than one explanation for free will beliefs might be seen in the general public. This is especially true in this study, as we assessed large representative samples in two different cultures. In order to assess whether libertarianism, compatibilism, or dualism offered the best explanation for free will beliefs, we performed an additional analysis.

In a first step, we tested which definition of free will (as assessed using the five FWI part2 items analyzed above: FWI2i1, FWI2i2, FWI2i5, FWI2i6, FWI2i7) showed the strongest relationship to FW-gen. For this purpose, we computed multiple linear models in the US sample, using the five FWI part2 items. We calculated a model fit (using the AIC, [35]) for each possible combination of variables, and then used a step-wise model selection procedure (using *stepAIC* from the *MASS* package) to determine the best-fitting model. This was done on both the main and the replication sample, and we only report effects that were consistently found in both independent samples. We then performed post-hoc tests (using *linearHypothesis* from the *car* package), using Bonferroni correction to account for multiple testing, in order to determine which FWI part2 item explained FW-gen the best. The same procedure was then performed on data from the SGP sample.

Similar to Analyses 1–3, we then also assessed behavior in part1 of the FWI directly. If we were to e.g. find that incompatibilist definitions of free will were most strongly related to FW-gen, we would expect FW-gen to be most strongly related to FW-de, and less so to FW-du. We therefore repeated the same model fitting and selection procedure as above, only now entering FW-de, FW-du, and their interaction as explanatory variables. We first determined the best-fitting model using the AIC as a criterion, and then performed post-hoc tests to determine which variable best explained FW-gen. All models in Analysis 4 were additionally estimated using a Bayesian framework (see S4 Analysis), in order to assess robustness of the results. Overall, these analyses identify statistical relationships between FW-gen and other FWI sub-scales. They demonstrate that e.g. FW-de is predictive of FW-gen, they do not demonstrate strict causal relationships.

## Results

### Descriptive statistics

**Fig 1:**
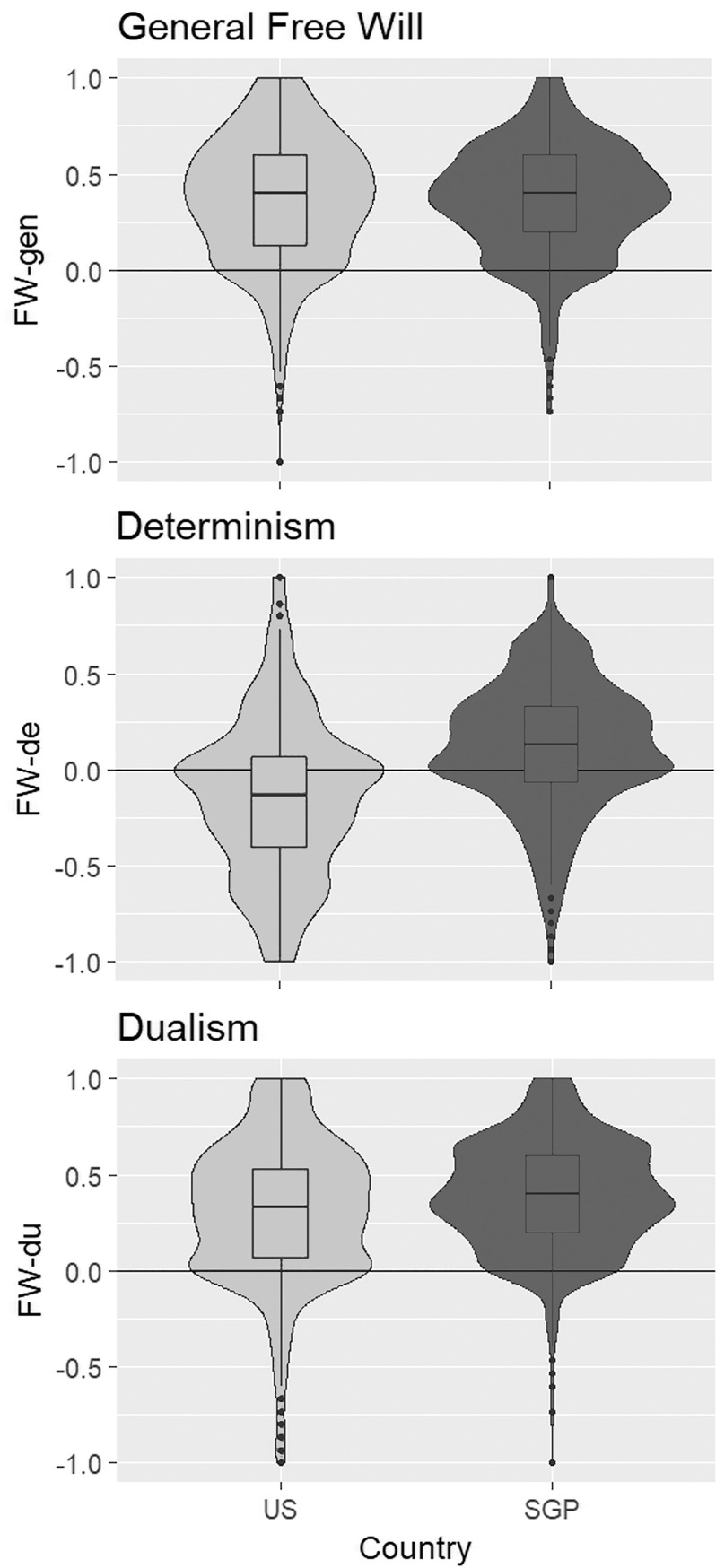
Free will beliefs. Violin / box plots depicting the distribution of general free will beliefs (FW-gen, above), determinism beliefs (FW-de, middle), and dualism beliefs (FW-du, below) in the United States (US, left/light grey) and Singapore (SGP, right/dark grey). Scores larger than 0 indicate a belief in e.g. determinism, while scores smaller than 0 indicate a disbelief in e.g. determinism. Scores of 0 indicate indifference.

First, we assessed the percentage of subjects believing in free will, determinism, and dualism (Fig 1). The FWI allows us to count how many subjects agree with beliefs according to its three dimensions. In the US, the majority did believe in free will (82.33%), and only a minority believed in determinism (30.77%). A vast majority of subjects also believed in dualism (75.77%). In SGP, the majority also believed in free will (85.44%), just like in the US. Interestingly, belief in determinism showed the opposite pattern in SGP as in the US, with the majority believing in it in SGP (59.00%). Dualism beliefs were even stronger than in the US, with a large majority believing in it (88.33%). This shows that across both cultures the majority largely agreed in their beliefs in free will and dualism, but had diverging views on determinism. We further assessed whether demographic variables (sex, age, education) affected any of the FWI sub-scale scores and found no effect on any sub-scale either in the US, or SGP (see S5 Analysis).

### Analysis 1: Libertarianism

As stated above, one major intuition that might be closely related to free will beliefs is libertarianism, the belief that we have free will because the physical world is not fully determined [14]. Agreement to central tenets of libertarian, incompatibilist theories is assessed in two items of part2 of the FWI (‘ability to do otherwise’, ‘unmoved mover’), and we first assessed agreement to these statements in the US and SGP. Most participants agreed with item 1 (‘ability to do otherwise’), in the US (76.6%), and in SGP (83.2%). We then tested whether agreement to this statement is related to FW-gen. We found it to be related positively in both the US, b = 0.10, R^2^ = 0.15, F(1,447) = 81.91, p < 0.001, BF_10_ > 150, and SGP, b = 0.11, R^2^ = 0.16, F(1,449) = 88.70, p < 0.001, BF_10_ > 150. Most participants also agreed to the ‘unmoved mover’ item, in the US (62%), and in SGP (70.6%). Again responses on this item were positively related to FW-gen in the US, b = 0.06, R^2^ = 0.05, F(1,449) = 27.24, p < 0.001, BF_10_ > 150, and SGP, b = 0.09, R^2^ = 0.13, F(1,449) = 66.60, p < 0.001, BF_10_ > 150, although the effect was slightly weaker than for item 1.

**Fig 2:**
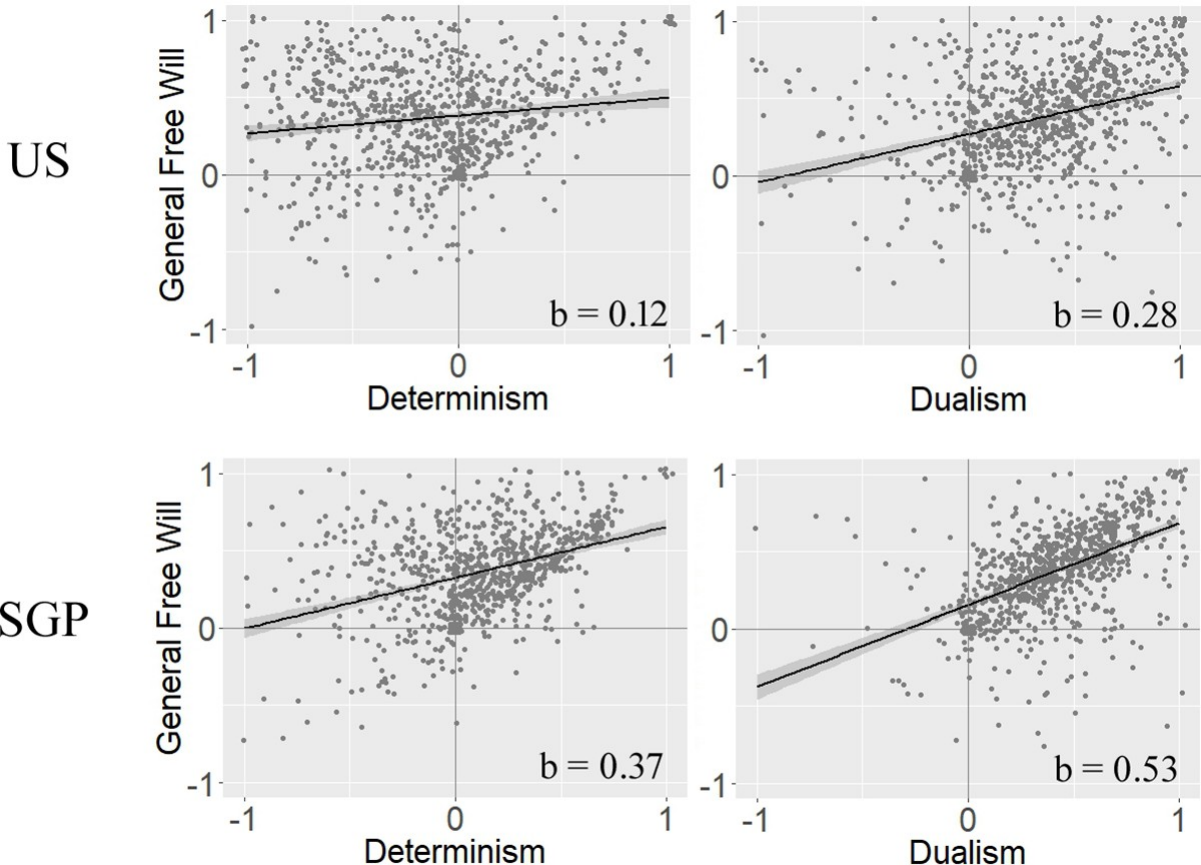
FWI sub-scale correlations. On the left, correlations between general free will beliefs and determinism are depicted, for the United States (US, n = 900, above) and Singapore (SGP, n = 900, below). On the right the relation between general free will beliefs and dualism is depicted. Each dot represents a single subject. Positive values represent belief in e.g. free will, negative values represent disbelief. For illustration purposes, the single subject data was slightly jittered to better depict its density. Black lines are fitted linear models, which are drawn with a 90% confidence interval (dark gray area) and the corresponding b value.

Based on these responses, one might expect that participants hold a libertarian view on free will, which suggest incompatibilism, i.e. the notion that free will cannot exist in a deterministic world. If participants held this view, this should be reflected in a negative relation of the FW-gen and FW-de scales. Counter to the prediction of a libertarian position, we found FW-gen and FW-de to be related positively in both the US, b = 0.12, R^2^ = 0.02, F(1,447) = 11.97, p < 0.001, BF_10_ = 32, and SGP, b = 0.37, R^2^ = 0.18, F(1,449) = 101.95, p < 0.001, BF_10_ > 150 (Fig 2), although the effect in the US was relatively small. This positive relation is more in line with compatibilist views on free will than with libertarian views (see Analysis 2).

The relation between FW-gen and FW-de can further be assessed through a cross-cultural comparison. If general free will beliefs were closely related to determinism beliefs, we should see a covariation of FW-gen and FW-de scores across cultures. We see a clear difference in FW-de, t(857) = -9.58, p < 0.001, d = 0.63, BF_10_ > 150, with US disbelieving in determinism and SGP believing in it (Fig 1). According to a libertarian view, we should observe the opposite effect for FW-gen, i.e. lower FW-gen scores in SGP than in the US. Yet, we found strong evidence for the absence of cultural differences in FW-gen, t(887) = -0.48, p = 0.63, d = 0.03, BF_10_ = 0.08. An additional control analysis demonstrated that these effects cannot be explained by mere differences in average age, sex-ratio, or level of educational between countries (see S6 Analysis). Thus, differences in determinism beliefs across cultures occur in the absence of any differences in general free will beliefs, again demonstrating that FW-gen and FW-de are not negatively related. Overall, this points to some inconsistencies in lay beliefs, as most participants agree to libertarian concepts of free will, yet still free will and determinism are positively related.

### Analysis 2: Compatibilism

Our previous analysis highlighted some of the inconsistencies linked to libertarianism as an explanation for pervasive general free will beliefs. An alternative explanation can be found in compatibilism, i.e. the view that free will and determinism are compatible and that one can believe in both at the same time. Again, we first assessed agreement to central concepts of compatibilist positions (‘choose according to beliefs/desires’, ‘no coercion’). Most participants agreed that free will means choosing according to one’s own beliefs and desires, US (57%), SGP (64%). However, these responses were only weakly related to FW-gen in SGP, b = 0.06, R^2^ = 0.06, F(1,449) = 28.08, p < 0.001, BF_10_ > 150, and unrelated in the US, b = 0.02, R^2^ = 0.008, F(1,447) = 3.85, p = 0.05, BF_10_ = 0.67. The ‘no coercion’ item showed somewhat different responses. In the US, participants mostly disagreed (37.2%) or were indifferent (36.5%), and in SGP participants mostly believed (49.3%) or were indifferent (36.7%). As before, responses were only weakly related to FW-gen in SGP, b = 0.07, R^2^ = 0.10, F(1,449) = 48.72, p < 0.001, BF_10_ > 150, and unrelated in the US, b = 0.005, R^2^ = 0.0007, F(1,447) = 0.31, p = 0.57, BF_10_ = 0.12.

These results show that agreement with compatibilist concepts of free will is higher in SGP, and participants in the US disagree with some central compatibilist claims. Based on this, we would expect subjects (especially in SGP) to show either no or a positive relation between FW-gen and FW-de, as compatibilists might (but do not have to) believe simultaneously in free will and determinism. In fact, we found FW-gen and FW-de to be related positively both in the US (b = 0.12, see Analysis 1), and in SGP (b = 0.37, Fig 2). Results in SGP show some consistency, in that the overall agreement to compatibilist concepts of free will was in line with their response patterns on part 1 of the FWI. Results in the US were less consistent however, as subjects only strongly agreed to libertarian concepts of free will, yet their response patterns on part 1 of the FWI were more in line with compatibilist theories.

### Analysis 3: Dualism

In the previous analyses, we focused on the relationship of FW-gen to FW-de and compatibilism. An alternative explanation might be that subjects believe in dualism, i.e. that the mind or soul is not a physical entity. This often entails that it is exempt from the deterministic laws that govern the physical world. One item of part 2 of the FWI explicitly assesses whether an immaterial soul is necessary for free will, and most participants agree that it is, US (77.1%), SGP (82,8%). Responses to this item were further positively related to FW-gen in the US, b = 0.06, R^2^ = 0.07, F(1,447) = 35.94, p < 0.001, BF_10_ > 150, and SGP, b = 0.10, R^2^ = 0.14, F(1,449) = 74.85, p < 0.001, BF_10_ > 150. Based on this result, one would further expect FW-gen and FW-du to be positively related. Indeed, we found a strong positive relationship in the US, b = 0.28, R^2^ = 0.10, F(1,447) = 54.60, p < 0.001, BF_10_ > 150, and in SGP, b = 0.53, R^2^ = 0.26, F(1,449) = 165.10, p < 0.001, BF_10_ > 150, where dualism alone explained about a quarter of the variance of FW-gen. This shows that people see dualism as a necessary condition for free will, and respond to part 1 of the FWI in a way that is consistent with this belief. As an additional exploratory analysis, we also assessed the relation of FW-de and FW-du (S7 Analysis).

### Analysis 4: Comparing different intuitions

**Fig 3:**
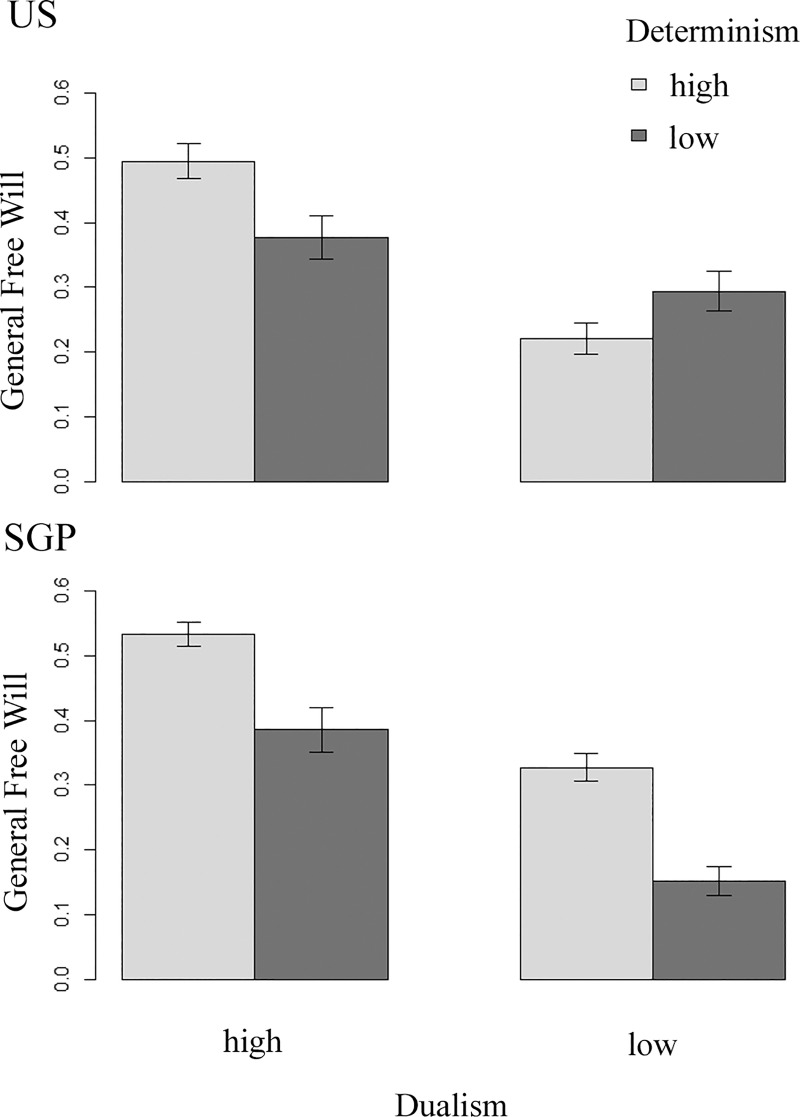
Effects of dualism and determinism on general free will beliefs. The effects of dualism and determinism on general free will beliefs are depicted for the US (above) and SGP (below). For visualization purposes, we performed a median split along both the dualism and determinism dimensions, showing data separately for high dualism beliefs (left), low dualism beliefs (right), high determinism beliefs (light gray), and low determinism beliefs (dark grey). Positive values indicate belief in free will, zero indicates indifference. Error bars represent SEM.

Given that psychological lay theories rarely are logically consistent [34], it is not entirely unexpected that our data provides evidence for multiple explanations. Yet, in order to systematically assess which theory provides a better prediction of general free will beliefs, we computed multiple different linear models and compared their fit to our data-set. First, we assessed which of the FWI part 2 items best explained FW-gen in the US. We found items 1 (‘ability to do otherwise’), 5 (‘necessity of immaterial soul’), & 6 (‘unmoved mover’) to be positively related to FW-gen, F(3,447) = 30.43, p < 0.001, R^2^ = 0.17, b_item1_ = 0.99, t(447) = 5.21, p < 0.001, b_item5_ = 0.59, t(447) = 3.05, p = 0.002, b_item6_ = 0.71, t(447) = 4.13, p < 0.001. Post-hoc tests revealed no significant differences between the three main effects, all Fs(1,447) < 1.70, ps > 0.19. Second, we assessed whether FW-de or FW-du is more strongly related to FW-gen. The winning model included both main effects and their interaction, F(3,447) = 30.6, p < 0.001, R^2^ = 0.16. We found only FW-du to be related to FW-gen, b_FW-du_ = 0.32, t(448) = 7.73, p < 0.001, but not FW-de, b_FW-de_ = 0.06, t(448) = 1.49, p = 0.13. Post-hoc tests revealed that FW-du is also more strongly related to FW-gen than FW-de is, F(1,448) = 19.48, p < 0.001. We further found a significant interaction, b_int_ = 0.03, t(447) = 4.46, p < 0.001. While determinism reduced FW-gen in subjects that believed the least in dualism, determinism increased FW-gen in subjects that believed the most in dualism (Fig 3). These results show that both libertarian and dualistic concepts are related to general FWB in the US, but that the link to dualism is stronger than the link to determinism.

In SGP, we found FWI part 2 items 1 (‘ability to do otherwise’), 5 (‘necessity of immaterial soul’), and 7 (‘no coercion’) to be related to FW-gen, F(3,445) = 58.00, p < 0.001, R^2^ = 0.28, b_item1_ = 1.25, t(445) = 7.16, p <0.001, b_item5_ = 0.85, t(445) = 4.96, p < 0.001, b_item7_ = 0.68, t(445) = 5.21, p < 0.001. Post-hoc testes revealed that of these items, only item 1 was found to be more strongly related to FW-gen than item 7, F(1,445) = 6.10, p = 0.01, while no difference was found between items 1 and 7, and items 5 and 7, all Fs (1,445) > 1.79, ps > 0.18. We also assessed the relation of FW-de and FW-du to FW-gen in the SGP sample. The winning model included both main effects, as well as their interaction, F(3,447) = 82.49, p < 0.001, R^2^ = 0.35. We found both a main effect of FW-de, b_FWI-det_ = 0.25, t(448) = 7.09, p < 0.001, and FW-du, b_FWI-du_ = 0.43, t(448) = 10.36, p < 0.001, as well as a significant interaction, b_int_ = 0.02, t(447) = 3.08, p = 0.002. Overall, determinism facilitated the positive effect of dualism on general free will beliefs, with this facilitation being stronger for subjects that believed relatively less in dualism than for subjects that believed relatively more in dualism (Fig 3). Post-hoc tests revealed that the effect of FW-du was significantly stronger than the effect of FW-de, F(1,448) = 8.11, p = 0.004. These results show that libertarian, compatibilist, and dualistic concepts are positively related to general free will beliefs, and that the link to dualism beliefs is stronger than the link to determinism beliefs. We also conducted a Bayesian version of Analysis 4 (S4 Analysis), which confirmed the results reported here.

Given that more than one intuition seems to predict free will beliefs, and that these views are not seen as mutually exclusive, we counted the number of compatibilist subjects that also believed dualism to be necessary for free will. We found a large majority to believe in both concepts in the US (86.00%) and SGP (89.56%). Additionally, most subjects believing in a libertarian version of free will also believed dualism to be necessary for free will (US 83.61%, SGP 91.32%). Paradoxically, we even found a majority of subjects that believed in a compatibilist definition of free will to also believe in an incompatibilist definition of free will (US 86.19%, SGP 92.29%), despite inherent logical inconsistencies of holding both beliefs simultaneously. Yet, despite this fact we also demonstrated that dualism was the single best predictor for general free will beliefs in both the US and SGP.

## Discussion

Here, we report results from a large, representative, cross-cultural online survey study examining free will beliefs in an individualistic western (US) and a collectivistic east Asian (SGP) culture [30]. First, we largely validated previous findings in larger, more representative samples [1], which is not at all common in psychology [32]. We also assessed our results using a novel analysis approach, investigating which of three prominent intuitions is most closely related to free will beliefs empirically: libertarianism, compatibilism, and dualism. Past research did not directly assess and compare these different theories, rather focusing on e.g. different sources of determinism beliefs [20]. Free will was at least partly explained by libertarian, compatibilist, and dualist theories, showing that more than one theory is prevalent in the general public. Contrary to our initial hypothesis, the link to dualist theories was the strongest and most consistent in our data-set.

### Free will and determinism

Whether we have free will or not has been debated amongst scholars for centuries, yet laypeople’s intuitions on this issue have only been studied fairly recently [23,36]. It has been pointed out that lay intuitions on free will are often conflicting [23], as we generally feel as if we have a free choice between multiple alternatives (free will), yet we also feel that everything that is happening should have a causal explanation (determinism). For many scholars, these intuitions are incompatible, motivating incompatibilist theories on free will, such as libertarianism [14], according to which we are free because our world is not deterministic, or hard determinism, according to which we have no free will because we are determined. Crucially, it remained unclear whether similar intuitions are prevalent in the general public as well. Our results highlight some inconsistencies of lay beliefs in the general public, by showing explicit agreement with libertarian concepts of free will (especially in the US) and simultaneously showing behavior that is more consistent with compatibilist theories. If participants behaved in a way that was consistent with their libertarian beliefs, we would have expected a negative relation between free will and determinism, but instead we saw a positive relation that is hard to reconcile with libertarian views (see also [3,18] for converging evidence) as well as hard determinist views (see S8 Analysis). This shows a disconnection between belief in explicit concepts regarding free will, and the resulting behavior, and future research should measure both independently.

Regarding determinism beliefs by themselves, previous work suggests that belief in determinism is relatively weak in the general public [23], but our data only partly support this notion. While most people in the US indeed did not believe in determinism, the opposite was true in Singapore, which underscores the need to study FWB in a variety of different cultures to avoid sampling biases [24]. One tentative explanation for this difference might be that the US are a highly individualistic country, while SGP is a more collectivistic country [30], and US citizens might be more strongly opposed to their behavior being determined by forces outside their direct control (see also [37] and S1 Analysis for a comparison of Locus of Control between countries). Although we have shown that this effect cannot be attributed to differences in age, sex, or education, the present data alone cannot conclusively attribute this effect to individualism alone. While individualism might be one of the most prominent differences between the US and SGP, both countries also differ along many other dimensions, which might potentially also explain this effect. Clearly, future work will have to address the link between individualism and free will beliefs more directly (see also [38]). Regarding intuitions on free will, belief in free will was pervasive in both cultures (see also [39]), and was correlated positively with determinism. Surprisingly, we found no difference in general free will beliefs across both countries, despite the high statistical power of our design. This shows the robustness of general free will beliefs despite the many differences between these two cultures. Overall, our results suggest that a large part of the general public has libertarian and compatibilist (SGP), or only libertarian (US) intuitions on free will, but behave in a way that is more in line with compatibilist intuitions irrespective whether they are from the US or SGP.

### Free will and dualism

Although dualistic beliefs are known to be pervasive (see [40–42] for previous evidence form smaller, more restricted samples), we were surprised by the *degree* to which both the general public in the US as well as SGP believed in mind-body dualism. The vast majority (more than 3 out of 4 participants) believes that the mind or soul is a non-physical entity, or at least that the mind cannot be reduced to the brain (see [20] for a discussion about different aspects of dualism beliefs). A similar proportion of subjects views an immaterial soul as a necessary condition for free will. Some previous work already demonstrated a high prevalence of dualism beliefs in a US sample [1], yet also demonstrated considerable inter-individual differences in dualism beliefs [43]. Here, we rather show that dualism beliefs are highly prevalent across different cultures, all age groups, both sexes, as well as different levels of education. Dualistic explanations of free will are not prominent in the current academic debate [44,45], but are strong in the general public. Previous empirical evidence in this issue was mixed. While some showed that dualism and FWB are independent [16,46], others found a relation of FWB and at least some aspects of dualism beliefs [20]. Our results demonstrate that, contrary to our initial hypotheses, free will beliefs are best predicted, and most closely related to dualism beliefs, and that this relation is stronger and more consistent than to compatibilist or libertarian intuitions. Dualism alone explained about a quarter of the FW-gen variance in SGP. One possible reason for this strong link might be religious convictions [43]. In the US, 95% believe in a life after death, which at least implicitly implies dualism [47], but see [48]. Clearly, future research will have to be directed at explaining why dualism is so highly prevalent in these countries and how exactly it is related to free will beliefs. Specifically, it will be highly interesting to differentiate between different aspects of dualism beliefs (see [20]) and assess such effects in representative cross-cultural samples as well. It will also be interesting to experimentally manipulate dualism beliefs and assess potential effects on FWB.

Despite the fact that dualism was most strongly related to general free will beliefs, the latter were at least partly explained by libertarian and compatibilist beliefs as well. Evidently, these theories are not mutually exclusive in the eyes of the general public [23]. It is possible that subjects believed in compatibilism and/or libertarianism *because* they view the mind as a non-physical entity. In line with this, we showed that the vast majority of compatibilists and libertarians were also dualist. Additionally, we found an interaction between determinism and dualism effects on general free will beliefs. In the US, determinism effects on FWB reverse depending on the level of dualism beliefs, and in SGP this effect is at least moderated in strength. Although the directionality of this effect will need to be tested more directly in future research, at the very least this is evidence for the complicated interaction of determinism and dualism beliefs and possible moderating effects of dualism beliefs. Yet, this key finding is somewhat at odds with the current philosophical debate surrounding free will, which focuses largely on its relation to determinism (e.g. [49]). Some argue for compatibilism [14], others for libertarianism [12] or hard determinism [10], but all of these theories try to explain how free will is related to determinism. Our results suggest that dualism is much more important to lay theories than it is for academic theories of free will, and determinism might not pose a threat to free will because of wide-spread dualistic beliefs (see also [50]).

These findings have further implications for studies trying to experimentally manipulate FWB [4,51,52]. In a typical experiment, participant might read a text that suggests we have no free will [52], which often also suggests materialism (e.g. only physical matter exists, and mental states are by-products of physical processes). Although it is possible that this manipulation directly reduces belief in free will, our results suggest a possible alternative explanation. Potentially, such manipulations reduce the belief in dualism instead, which is generally seen as a necessary condition for free will (see Analysis 3). In this case, such a manipulation might lead to a reduction in free will beliefs, but crucially moderated by dualism beliefs. Future experimental research should investigate which specific beliefs are directly affected by FWB manipulations.

### Conclusion

We have shown that free will beliefs in the general public are most closely related to a strong belief in dualism. This was true in different cultures, age groups, and levels of education. As noted in the beginning, recent neuroscientific findings have been taken to suggest that our choices might originate from unconscious brain activity [8,9], but see [53], which has led some to predict an erosion of free will beliefs with potentially serious consequences for our sense of responsibility and even the criminal justice system [7]. However, even if neuroscience were to fully describe and explain the causal chain of processes in the physical brain, this need not lead to an erosion of free will beliefs in the general public. Although some might indeed see this as a threat to free will (US citizens with low dualism beliefs), most will not likely because of a wide-spread belief in dualism (see also [21]). Our findings also highlight the need for cross-cultural examinations of free will beliefs and related constructs, as previous findings from (mostly undergraduate) US samples do not fully generalize to other cultures.

## Supporting information

S1 Analysis Locus of control. (PDF)

S2 Analysis Validating the FWI. (PDF)

S3 Analysis Acquiescence bias in the FWI. (PDF)

S4 Analysis Bayesian model comparison. (PDF)

S5 Analysis Effect of demographic variables on free will beliefs. (PDF)

S6 Analysis Cultural differences in sex-, age- and education-matched samples. (PDF)

S7 Analysis Relation of determinism and dualism. (PDF)

S8 Analysis Hard determinism. (PDF)

## References

[1] Nadelhoffer T, Shepard J, Nahmias E, Sripada C, Ross LT. The free will inventory: Measuring beliefs about agency and responsibility. Conscious Cogn. 2014;25: 27–41. 10.1016/j.concog.2014.01.006 24561311

[2] Paulhus DL, Carey JM. The FAD–Plus: Measuring Lay Beliefs Regarding Free Will and Related Constructs. J Pers Assess. 2011;93: 96–104. 10.1080/00223891.2010.528483 21184335

[3] Nahmias E, Morris S, Nadelhoffer T, Turner J. Surveying Freedom: Folk Intuitions about free will and moral responsibility. Philos Psychol. 2005;18: 561–584. 10.1080/09515080500264180

[4] Baumeister RF, Masicampo EJ, DeWall CN. Prosocial Benefits of Feeling Free: Disbelief in Free Will Increases Aggression and Reduces Helpfulness. Pers Soc Psychol Bull. 2009;35: 260–268. 10.1177/0146167208327217 19141628

[5] Feldman G. Making sense of agency: Belief in free will as a unique and important construct. Soc Personal Psychol Compass. 2017;11: e12293 10.1111/spc3.12293

[6] Earp BD, Everett JA, Nadelhoffer T, Caruso GD, Shariff A, Sinnott-Armstrong W. Determined to Be Humble? Exploring the Relationship Between Belief in Free Will and Humility. 2018; 10.31234/osf.io/3bxra

[7] Greene J, Cohen J. For the law, neuroscience changes nothing and everything. Philos Trans R Soc Lond B Biol Sci. 2004;359: 1775–1785. 10.1098/rstb.2004.1546 15590618 PMC1693457

[8] Libet B, Gleason CA, Wright EW, Pearl DK. Time of conscious intention to act in relation to onset of cerebral activity (readiness-potential) the unconscious initiation of a freely voluntary act. Brain. 1983;106: 623–642. 10.1093/brain/106.3.623 6640273

[9] Soon CS, Brass M, Heinze H-J, Haynes J-D. Unconscious determinants of free decisions in the human brain. Nat Neurosci. 2008;11: 543–545. 10.1038/nn.2112 18408715

[10] Wegner DM. The mind’s best trick: how we experience conscious will. Trends Cogn Sci. 2003;7: 65–69. 10.1016/S1364-6613(03)00002-0 12584024

[11] Harris S. Free Will. Simon and Schuster; 2012.

[12] Fischer JM, Kane R, Pereboom D, Vargas M. Four Views on Free Will. Wiley-Blackwell; 2007.

[13] Rolnick J, Parvizi J. Automatisms: Bridging clinical neurology with criminal law. Epilepsy Behav. 2011;20: 423–427. 10.1016/j.yebeh.2010.09.033 21145287

[14] Kane RH. Agency, Responsibility, and Indeterminism: Reflections on Libertarian Theories of Free Will In: Honderich T, editor. Freedom and Determinism. Bradford Book/MIT Press; 2004.

[15] Mele A. Free Will and Substance Dualism: The Real Scientific Threat to Free Will? In: Sinnot-Armstrong W, editor. Moral Psychology, Vol 4: Free Will and Responsibility. MIT Press; 2014.

[16] Monroe AE, Malle BF. From Uncaused Will to Conscious Choice: The Need to Study, Not Speculate About People’s Folk Concept of Free Will. Rev Philos Psychol. 2010;1: 211–224. 10.1007/s13164-009-0010-7

[17] Stillman TF, Baumeister RF, Mele AR. Free will in everyday life: Autobiographical accounts of free and unfree actions. Philos Psychol. 2011;24: 381–394. 10.1080/09515089.2011.556607

[18] Nahmias E, Morris SG, Nadelhoffer T, Turner J. Is Incompatibilism Intuitive? Philos Phenomenol Res. 2006;73: 28–53. 10.1111/j.1933-1592.2006.tb00603.x

[19] Deery O, Davis T, Carey J. The Free-Will Intuitions Scale and the question of natural compatibilism. Philos Psychol. 2015;28: 776–801. 10.1080/09515089.2014.893868

[20] Forstmann M, Burgmer P. A free will needs a free mind: Belief in substance dualism and reductive physicalism differentially predict belief in free will and determinism. Conscious Cogn. 2018;63: 280–293. 10.1016/j.concog.2018.07.003 30001841

[21] Nahmias E, Shepard J, Reuter S. It’s OK if ‘my brain made me do it’: People’s intuitions about free will and neuroscientific prediction. Cognition. 2014;133: 502–516. 10.1016/j.cognition.2014.07.009 25195077

[22] Preston JL, Ritter RS, Hepler J. Neuroscience and the soul: Competing explanations for the human experience. Cognition. 2013;127: 31–37. 10.1016/j.cognition.2012.12.003 23318352

[23] Nichols S. Experimental Philosophy and the Problem of Free Will. Science. 2011;331: 1401–1403. 10.1126/science.1192931 21415346

[24] Henrich J, Heine SJ, Norenzayan A. The weirdest people in the world? Behav Brain Sci. 2010;33: 61–83. 10.1017/S0140525X0999152X 20550733

[25] Bloom P. My Brain Made Me Do It. J Cogn Cult. 2006;6: 209–214. 10.1163/156853706776931303

[26] Ewusi-Boisvert E, Racine E. A Critical Review of Methodologies and Results in Recent Research on Belief in Free Will. Neuroethics. 2018;11: 97–110. 10.1007/s12152-017-9346-3

[27] Martin ND, Rigoni D, Vohs KD. Free will beliefs predict attitudes toward unethical behavior and criminal punishment. Proc Natl Acad Sci. 2017;114: 7325–7330. 10.1073/pnas.1702119114 28652361 PMC5514725

[28] Hui C-CH. Locus of control: A review of cross-cultural research. Int J Intercult Relat. 1982;6: 301–323. 10.1016/0147-1767(82)90036-0

[29] Smith PB, Trompenaars F, Dugan S. The Rotter Locus of Control Scale in 43 Countries: A Test of Cultural Relativity. Int J Psychol. 1995;30: 377–400. 10.1080/00207599508246576

[30] Hofstede G, Hofstede GJ, Minkov M. Cultures and Organizations: Software of the Mind, Third Edition McGraw Hill Professional; 2010.

[31] Hofstede GH, Hofstede G. Culture’s Consequences: Comparing Values, Behaviors, Institutions and Organizations Across Nations. SAGE; 2001.

[32] Collaboration OS. Estimating the reproducibility of psychological science. Science. 2015;349: aac4716 10.1126/science.aac4716 26315443

[33] Wagenmakers E-J. A practical solution to the pervasive problems of p values. Psychon Bull Rev. 2007;14: 779–804. 10.3758/BF03194105 18087943

[34] Nichols S. The Folk Psychology of Free Will: Fits and Starts. Mind Lang. 2004;19: 473–502. 10.1111/j.0268-1064.2004.00269.x

[35] Burnham KP, Anderson DR. Multimodel Inference: Understanding AIC and BIC in Model Selection. Sociol Methods Res. 2004;33: 261–304. 10.1177/0049124104268644

[36] Sosa E. Experimental philosophy and philosophical intuition. Philos Stud. 2007;132: 99–107. 10.1007/s11098-006-9050-3

[37] Vignoles VL, Owe E, Becker M, Smith PB, Easterbrook MJ, Brown R, et al Beyond the ‘east–west’ dichotomy: Global variation in cultural models of selfhood. J Exp Psychol Gen. 2016;145: 966–1000. 10.1037/xge0000175 27359126

[38] Feldman G, Farh J-L, Wong KFE. Agency Beliefs Over Time and Across Cultures: Free Will Beliefs Predict Higher Job Satisfaction. Pers Soc Psychol Bull. 2018;44: 304–317. 10.1177/0146167217739261 29191084 PMC5810915

[39] Sarkissian H, Chatterjee A, Brigard FD, Knobe J, Nichols S, Sirker S. Is Belief in Free Will a Cultural Universal? Mind Lang. 2010;25: 346–358. 10.1111/j.1468-0017.2010.01393.x

[40] Demertzi A, Liew C, Ledoux D, Bruno M-A, Sharpe M, Laureys S, et al Dualism persists in the science of mind. Ann N Y Acad Sci. 2009;1157: 1–9. 10.1111/j.1749-6632.2008.04117.x 19351351

[41] Forstmann M, Burgmer P. Adults are intuitive mind-body dualists. J Exp Psychol Gen. 2015;144: 222–235. 10.1037/xge0000045 25494547

[42] Miresco MJ, Kirmayer LJ. The Persistence of Mind-Brain Dualism in Psychiatric Reasoning About Clinical Scenarios. Am J Psychiatry. 2006;163: 913–918. 10.1176/ajp.2006.163.5.913 16648335

[43] Lindeman M, Riekki T, Svedholm-Häkkinen AM. Individual Differences in Conceptions of Soul, Mind, and Brain. J Individ Differ. 2015;36: 157–162. 10.1027/1614-0001/a000167

[44] Robbins P, Jack AI. The phenomenal stance. Philos Stud. 2006;127: 59–85. 10.1007/s11098-005-1730-x

[45] Churchland PS. The Hornswoggle Problem. J Conscious Stud. 1996;3: 402–8.

[46] Forstmann M, Burgmer P, Mussweiler T. “The Mind Is Willing, but the Flesh Is Weak”: The Effects of Mind-Body Dualism on Health Behavior. Psychol Sci. 2012;23: 1239–1245. 10.1177/0956797612442392 22972908

[47] Bering JM. The folk psychology of souls. Behav Brain Sci. 2006;29: 453–462. 10.1017/S0140525X06009101 17156519

[48] Hodge KM. Descartes’ Mistake: How Afterlife Beliefs Challenge the Assumption that Humans are Intuitive Cartesian Substance Dualists. J Cogn Cult. 2008;8: 387–415. 10.1163/156853708X358236

[49] Feldman G, Chandrashekar SP. Laypersons’ Beliefs and Intuitions About Free Will and Determinism: New Insights Linking the Social Psychology and Experimental Philosophy Paradigms. Soc Psychol Personal Sci. 2018;9: 539–549. 10.1177/1948550617713254 30220960 PMC6113710

[50] Unger P. Free Will and Scientiphicalism. Philos Phenomenol Res. 2002;65: 1–25. 10.1111/j.1933-1592.2002.tb00180.x

[51] Crone DL, Levy NL. Are Free Will Believers Nicer People? (Four Studies Suggest Not). Soc Psychol Personal Sci. 2019;10: 612–619. 10.1177/1948550618780732 31249653 PMC6542011

[52] Rigoni D, Kühn S, Gaudino G, Sartori G, Brass M. Reducing self-control by weakening belief in free will. Conscious Cogn. 2012;21: 1482–1490. 10.1016/j.concog.2012.04.004 22579497

[53] Schultze-Kraft M, Birman D, Rusconi M, Allefeld C, Görgen K, Dähne S, et al The point of no return in vetoing self-initiated movements. Proc Natl Acad Sci. 2016;113: 1080–1085. 10.1073/pnas.1513569112 26668390 PMC4743787

