# Supplementary material for: Free will beliefs are better predicted by dualism than determinism beliefs across different cultures
Source: PLoS One. 2019 Sep 11;14(9):e0221617. doi: 10.1371/journal.pone.0221617 (PMC6738589; doi:10.1371/journal.pone.0221617)
Supplement: S1 Analysis — (PDF) [file pone.0221617.s001.pdf]

S1 Analysis: Locus of control

Our main hypothesis was that we expected cross-cultural differences in FWB as locus of control (LOC) was found to vary across individualistic / collectivistic cultures (1) and LOC was found to be correlated with FWB (2). We tested these assumptions by first assessing cross-cultural differences in LOC using a Bayesian two-sample t-test comparing internal LOC, external chance LOC, and external powerful others LOC between the US and SGP. We found no sufficient evidence for a cross-cultural difference in internal locus of control, $d = 0.17$, $BF10 = 2.57$, but found strong evidence for a stronger external locus of control (both chance and powerful others) in SGP, as compared to the US, chance, $d = 0.50$, $BF10 > 150$, powerful others, $d = 0.47$, $BF10 > 150$. Overall, these results demonstrate that locus of control does indeed show the expected cross-cultural differences, with external locus of control being stronger in SGP than in US. Results for internal locus of control are inconclusive despite the large sample size used in this study. Numerically, they point towards stronger internal locus of control in the US.

In the next step we then tested whether locus of control was related to free will beliefs by computing pairwise correlations of each sub-scale of these two questionnaires (Supplementary Figure 1). Locus of control and free will beliefs are correlated in our data-set. In the US, we found general free will beliefs to be mostly related to internal locus of control ($r = 0.47$), and found determinisms to be mostly related to external locus of control (chance $r = 0.60$, powerful others = 0.47). In SGP we found a similar pattern of results. General free will beliefs were mostly related to internal locus of control ($r =0.44$), and determinism beliefs were mostly related to external locus of control (chance $r = 0.46$, powerful others $r = 0.38$). This demonstrates that free will beliefs and locus of control are indeed related in our data-set, thus confirming our initial reasoning as to why one might expect differences in free will beliefs between the US and SGP.

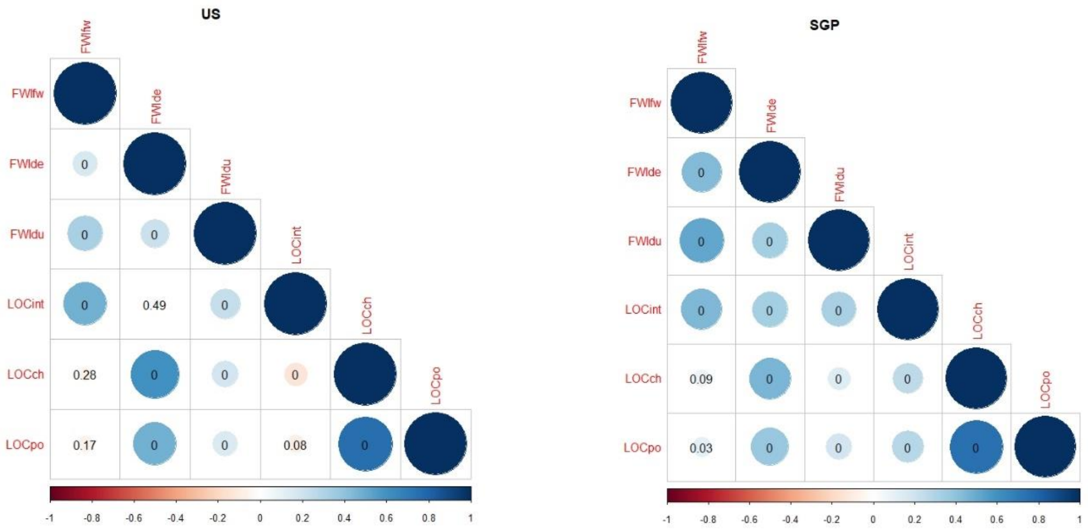

Supplementary Figure 1: Correlations of LOC and FWI sub-scales. FWIfw = general free will, FWIde = determinism, FWIdu = dualism, LOCint = internal, LOCch = chance, LOCpo = powerful others. Color and circle size represent the strength of the correlation. In-cell numbers represent the p-values of a significance test vs 0.

1. Hui C-CH. Locus of control: A review of cross-cultural research. Int J Intercult Relat. 1982 Jan 1;6(3):301–23.

2. Paulhus DL, Carey JM. The FAD–Plus: Measuring Lay Beliefs Regarding Free Will and Related Constructs. J Pers Assess. 2011 Jan 1;93(1):96–104.
