# Supplementary material for: Free will beliefs are better predicted by dualism than determinism beliefs across different cultures
Source: PLoS One. 2019 Sep 11;14(9):e0221617. doi: 10.1371/journal.pone.0221617 (PMC6738589; doi:10.1371/journal.pone.0221617)
Supplement: S2 Analysis — (PDF) [file pone.0221617.s002.pdf]

S2 Analysis: Validating the FWI

In order to validate the FWI as a measure for free will beliefs we performed confirmatory factor analyses, and calculated internal consistency measures, separately for the US and SGP. We first performed a confirmatory factor analysis (using the *lavaan* package) on the US sample, using the 3-factor structure proposed by (1). Model fit was assessed using GFI, CFI, AGFI, and RMSEA. The overall model fit was: GFI (.897), CFI (.886), AGFI (.858), RMSEA (.090). The model thus fits the data moderately well, although the fit is slightly worse than in the original paper, especially in measures taking sample size into account (RMSEA). Internal consistencies of the three sub-scales (computed using *cohensD* from the *lsr* package) were satisfactory: FW-gen ($\alpha = .81$), FW-de ($\alpha = .85$), FW-du ($\alpha = .82$). Results for the SGP sample were broadly similar. The overall fit of the 3-factor model was GFI (.909), CFI (.883), AGFI (.874), RMSEA (.083). The internal consistencies were FW-gen ($\alpha = .79$), FW-de ($\alpha = .81$), FW-du ($\alpha = .78$). Overall, the factor structure of the FWI could be confirmed in both samples.

1. Nadelhoffer T, Shepard J, Nahmias E, Sripada C, Ross LT. The free will inventory: Measuring beliefs about agency and responsibility. Conscious Cogn. 2014 Apr;25:27–41.
