# Supplementary material for: Free will beliefs are better predicted by dualism than determinism beliefs across different cultures
Source: PLoS One. 2019 Sep 11;14(9):e0221617. doi: 10.1371/journal.pone.0221617 (PMC6738589; doi:10.1371/journal.pone.0221617)
Supplement: S3 Analysis — (PDF) [file pone.0221617.s003.pdf]

S3 Analysis: Acquiescence bias in the FWI

Given that the FWI has no reverse-coded items, subjects might exhibit acquiescence bias. In order to control for this potential bias, we analyzed data from an unrelated questionnaire for which data was acquired from the same subjects: the self-efficacy scale (SES, (1). On this scale 50% of all items are reverse coded. In order to assess acquiescence bias, we correlated positively and reverse-coded items. If we found positive correlations between those items, this would show acquiescence bias. However, our results show clear negative correlations (Supplementary Figure 3), ruling out acquiescence bias in our samples.

US SGP

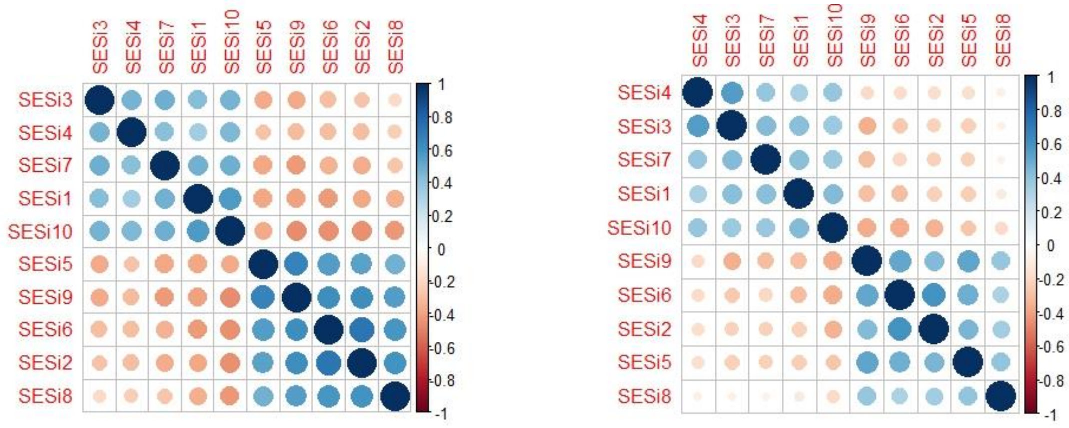

Supplementary Figure 3: Acquiescence bias. correlation matrix of SES items for SGP and US. A clear negative correlation can be seen between positively coded items (1,3,4,7,10) and reverse-coded items (2,5,6,8,9). SESi3 = self-efficacy scale item 3, etc. Blue colors indicate positive, red colors negative correlations. Circle sizes indicate the strength of the correlation. US = United States, SGP = Singapore.

1. Schwarzer R, Bäßler J, Kwiatek P, Schröder K, Zhang JX. The Assessment of Optimistic Self-beliefs: Comparison of the German, Spanish, and Chinese Versions of the General Self-efficacy Scale. Appl Psychol. 1997 Jan 1;46(1):69–88.
