# Supplementary material for: Free will beliefs are better predicted by dualism than determinism beliefs across different cultures
Source: PLoS One. 2019 Sep 11;14(9):e0221617. doi: 10.1371/journal.pone.0221617 (PMC6738589; doi:10.1371/journal.pone.0221617)
Supplement: S4 Analysis — (PDF) [file pone.0221617.s004.pdf]

S4 Analysis: Bayesian model comparison

To assess the robustness of our model selection procedure, we repeated Analysis 4, only in a Bayesian framework (using *lmBF* from the *BayesFactor* package). The analyses performed were identical to the one's reported in the main body in all other respects. Model-fit and model selection was now performed by computing Bayes Factors (BF_{10}) for each model. The model with the highest BF_{10} was selected as the best-fitting. Given that BF_{10}s are transitive, we were also able to assess relative evidence for a specific model, as compared to a different model.

In the US, we found FWI part2 items 1, 5, and 6 to best explain FW-gen ($BF_{10} > 150$, Supplementary Table 4.1). We further found item 1 to explain FW-gen better than item 5 ($BF_{10} = 133.44$) and item 6 ($BF_{10} = 119.69$). In SGP, we found FW-gen to be best explained by FWI part2 items 1, 5, and 7 ($BF_{10} > 150$, Supplementary Table 4.2), and found item 1 to better explain FW-gen than item 5 ($BF_{10} > 150$), and item 7 ($BF_{10} > 150$). Thus, believing that free will is 'the ability to do otherwise' is most predictive of general free will beliefs in the US and SGP.

In the second part of the analysis, we predicted FW-gen from determinism and/or dualism, and found it best predicted by a model containing both main effects and their interaction in the US ($BF_{10} > 150$, Supplementary Table 4.3) and SGP ($BF_{10} > 150$, Supplementary Table 4.4). We found dualism to better predict FW-gen than determinism in both the US ($BF_{10} > 150$) and SGP ($BF_{10} > 150$).

In sum, our Bayesian model selection procedure replicates our initial findings, demonstrating their robustness.

Supplementary Table 4.1: Predicting FW-gen from FWI part2 items in the US

Predictors	BF10
FWIfw~ FWI2i1	1123350414.6369011
FWIfw~ FWI2i2	6.0325513
FWIfw~ FWI2i5	8418605.5172399
FWIfw~ FWI2i6	9385164.5131354
FWIfw~ FWI2i7	0.1217871
FWIfw~ FWI2i1+FWI2i2	397088996.1063571
FWIfw~ FWI2i1+FWI2i5	1689290813265.9353027
FWIfw~ FWI2i1+FWI2i6	66822153500982.3437500
FWIfw~ FWI2i1+FWI2i7	140211750.0237993
FWIfw~ FWI2i2+FWI2i5	3759990.8834416
FWIfw~ FWI2i2+FWI2i6	6057392.8327130
FWIfw~ FWI2i2+FWI2i7	0.9266511
FWIfw~ FWI2i5+FWI2i6	15231208568.9130821
FWIfw~ FWI2i5+FWI2i7	1086560.9043294
FWIfw~ FWI2i6+FWI2i7	1291234.3852653
FWIfw~ FWI2i1+FWI2i2+FWI2i5	312004991864.6317139
FWIfw~ FWI2i1+FWI2i2+FWI2i6	11553455795778.7539062
FWIfw~ FWI2i1+FWI2i2+FWI2i7	60357087.8252611
FWIfw~ FWI2i1+FWI2i5+FWI2i6	800438358417516.6250000
FWIfw~ FWI2i1+FWI2i5+FWI2i7	237252459644.4650574
FWIfw~ FWI2i1+FWI2i6+FWI2i7	9179193381900.0019531
FWIfw~ FWI2i2+FWI2i5+FWI2i6	3959227299.7309098
FWIfw~ FWI2i2+FWI2i5+FWI2i7	600046.5095521
FWIfw~ FWI2i2+FWI2i6+FWI2i7	968847.9424766
FWIfw~ FWI2i5+FWI2i6+FWI2i7	2250617477.8767490
FWIfw~ FWI2i1+FWI2i2+FWI2i5+FWI2i6	128944290946617.6718750
FWIfw~ FWI2i1+FWI2i2+FWI2i5+FWI2i7	51581340539.5074539
FWIfw~ FWI2i1+FWI2i2+FWI2i6+FWI2i7	1844539377022.8972168
FWIfw~ FWI2i1+FWI2i5+FWI2i6+FWI2i7	123320946567499.0937500
FWIfw~ FWI2i2+FWI2i5+FWI2i6+FWI2i7	677653014.0191405
FWIfw~ FWI2i1+FWI2i2+FWI2i5+FWI2i6+FWI2i7	22457289590466.1289062

Depicted are the different models tested, which all predicted general free will beliefs (FWIfw) from different FWI part2 items (e.g. FWI2i1). On the right side you see the corresponding BF10. The winning model is highlighted. Formulas are printed in R syntax.

Supplementary Table 4.2: Predicting FW-gen from FWI part2 items in SGP

Predictors	BF10
FWIfw~ FWI2i1	27381637562046644224.000000
FWIfw~ FWI2i2	8.404057
FWIfw~ FWI2i5	26433903526949.085937
FWIfw~ FWI2i6	51165911.143206
FWIfw~ FWI2i7	3889012.058981
FWIfw~ FWI2i1+FWI2i2	3234375618591420416.000000
FWIfw~ FWI2i1+FWI2i5	621060650875733537728882.000000
FWIfw~ FWI2i1+FWI2i6	1340809892176336837628.000000
FWIfw~ FWI2i1+FWI2i7	20225536745384751262426 82.000000
FWIfw~ FWI2i2+FWI2i5	11045259127046.396484
FWIfw~ FWI2i2+FWI2i6	17390141.693585
FWIfw~ FWI2i2+FWI2i7	1468406.198672
FWIfw~ FWI2i5+FWI2i6	365473677542638.375000
FWIfw~ FWI2i5+FWI2i7	114145071349464555 52.000000
FWIfw~ FWI2i6+FWI2i7	19653537417.030762
FWIfw~ FWI2i1+FWI2i2+FWI2i5	72313449219659560658484.000000
FWIfw~ FWI2i1+FWI2i2+FWI2i6	162429977432526710378 4.000000
FWIfw~ FWI2i1+FWI2i2+FWI2i7	29861814979570417690604 4.000000
FWIfw~ FWI2i1+FWI2i5+FWI2i6	129663973287563611874648 0.000000
FWIfw~ FWI2i1+FWI2i5+FWI2i7	29146651950092751192044024024.000000
FWIfw~ FWI2i1+FWI2i6+FWI2i7	615775886549215837440068 0.000000
FWIfw~ FWI2i2+FWI2i5+FWI2i6	82657029420507.953125
FWIfw~ FWI2i2+FWI2i5+FWI2i7	1448277565613899520.000000
FWIfw~ FWI2i2+FWI2i6+FWI2i7	3543539062.128849
FWIfw~ FWI2i5+FWI2i6+FWI2i7	3165151725508921856.000000
FWIfw~ FWI2i1+FWI2i2+FWI2i5+FWI2i6	17792834948333830983406 8.000000
FWIfw~ FWI2i1+FWI2i2+FWI2i5+FWI2i7	6809424782990103755626282046.000000
FWIfw~ FWI2i1+FWI2i2+FWI2i6+FWI2i7	1435554538957411788564842.000000
FWIfw~ FWI2i1+FWI2i5+FWI2i6+FWI2i7	4637858343847633723206420208.000000
FWIfw~ FWI2i2+FWI2i5+FWI2i6+FWI2i7	458825439500677504.000000
FWIfw~ FWI2i1+FWI2i2+FWI2i5+FWI2i6+FWI2i7	13468911072290891004284464 62.000000

Supplementary Table 4.3: Predicting FW-gen from FW-de and/or FW-du in US

Predictors	BF10
FWIfw~ FWIde	5.290966
FWIfw~ FWIdu	1620298167685.960938
FWIfw~ FWIde+FWIdu	539472681861.289612
FWIfw~ FWIde*FWIdu	986065222197661.500000

Supplementary Table 4.4: Predicting FW-gen from FW-de and/or FW-du in SGP

Predictors	Predictors
FWIfw~ FWIde	36409050702
FWIfw~ FWIdu	18127716707060409479488888880024
FWIfw~ FWIde+FWIdu	1196475447488963031868884444848226 84
FWIfw~ FWIde*FWIdu	77057287028242333272646020024666604 88
