# Supplementary material for: Free will beliefs are better predicted by dualism than determinism beliefs across different cultures
Source: PLoS One. 2019 Sep 11;14(9):e0221617. doi: 10.1371/journal.pone.0221617 (PMC6738589; doi:10.1371/journal.pone.0221617)
Supplement: S5 Analysis — (PDF) [file pone.0221617.s005.pdf]

S5 Analysis: Effects of demographic variables on free will beliefs

In order to assess the effect of demographic variables (age, sex, years of education) on FWI ratings, we estimated several linear models, separately predicting each of the three FWI sub-scales from age, sex, and years of education (using *lmBF* from the *BayesFactor* package). In the US, we found that none of these variables predicted FW-gen, FW-de, or FW-du (all BF_{10}s < 1.19). The same was true in SGP (all BF_{10}s < 0.47). Demographic variables thus have negligible effects on general free will, determinism, and dualism beliefs.
