# Supplementary material for: Free will beliefs are better predicted by dualism than determinism beliefs across different cultures
Source: PLoS One. 2019 Sep 11;14(9):e0221617. doi: 10.1371/journal.pone.0221617 (PMC6738589; doi:10.1371/journal.pone.0221617)
Supplement: S6 Analysis — (PDF) [file pone.0221617.s006.pdf]

S6 Analysis: Cultural differences in sex-, age- and education-matched samples.

When comparing FWB across cultures, differences might stem from cultural differences, or merely from differences in age or education, given that our SGP sample (mean age = 38.85 years, mean years of education = 15.38) was both younger and had a longer education than the US sample (mean age = 41.08 years, mean years of education = 14.12). In order to rule out such effects we performed an additional control analysis. We first matched both the US and SGP samples with respect to age, sex, and education. This was done by randomly down-sampling both samples until they matched in sex-ratio, age, and education. For instance, there were n=140 18-24 year olds in the SGP sample, but only n=117 18-24 year olds in the US sample. We thus randomly selected n=117 18-24 year olds in the SGP sample, and removed all other participants in that age group. After having thus matched both samples, we still found FW-de to be stronger in SGP than in the US, $t(1507) = -12.874$, $p < 0.001$, $d = 0.65$, $BF_{10} > 150$, but found no difference in FW-gen, $t(1542) = 0.50$, $p = 0.62$, $d = 0.03$, $BF_{10} = 0.06$, matching our original results. Thus, cultural differences cannot be merely explained by either age, sex, or education.
