# Supplementary material for: Free will beliefs are better predicted by dualism than determinism beliefs across different cultures
Source: PLoS One. 2019 Sep 11;14(9):e0221617. doi: 10.1371/journal.pone.0221617 (PMC6738589; doi:10.1371/journal.pone.0221617)
Supplement: S7 Analysis — (PDF) [file pone.0221617.s007.pdf]

S7 Analysis: Relation of determinism and dualism

Some have argued that determinism and dualism beliefs are unrelated (1), while others found them to be positively related (2). Here, we also estimated correlations in a Bayesian framework, reporting the correlation coefficient, the probability of it being above or below zero ($p(r>0)$, $p(r>0)$), and 95% credible intervals (95% CI), which indicates the range of values within which the correlation falls with a 95% probability. If this interval did not include 0, we interpreted the correlation as either positive or negative. Classically estimated (frequentist) correlation coefficients are also reported for the interested reader. We found FW-de and FW-du to be positively correlated in both the US, $r = 0.21$, 95%CI = [0.11, 0.30], $p(r>0) > 0.99$, (classical estimation: $r = 0.17$, $p < 0.001$), and SGP, $r = 0.35$, 95%CI = [0.26, 0.42], $p(r>0) > 0.99$, (classical estimation: $r = 0.34$, $p < 0.001$). Our results thus suggest a positive relation between determinism and dualism beliefs.

1. Nadelhoffer T, Shepard J, Nahmias E, Sripada C, Ross LT. The free will inventory: Measuring beliefs about agency and responsibility. Conscious Cogn. 2014 Apr;25:27–41.

2. Forstmann M, Burgmer P. A free will needs a free mind: Belief in substance dualism and reductive physicalism differentially predict belief in free will and determinism. Conscious Cogn. 2018 Aug 1;63:280–93.
