# Supplementary material for: Free will beliefs are better predicted by dualism than determinism beliefs across different cultures
Source: PLoS One. 2019 Sep 11;14(9):e0221617. doi: 10.1371/journal.pone.0221617 (PMC6738589; doi:10.1371/journal.pone.0221617)
Supplement: S8 Analysis — (PDF) [file pone.0221617.s008.pdf]

S8 Analysis: Hard determinism

One prominent theory on free will especially amongst (neuro)scientists is hard determinism, the notion that we do not have free will because the physical world is fully determined and the mind is a physical entity. A hard determinist would answer the FWI by disbelieving in FW-gen and FW-du, but believing to FW-de. We counted the number of subjects showing such a response pattern, and only found a minute fraction to respond in accordance with this theory in the US (0.3%, n = 3), and no subject in SGP (0%, n = 0). This demonstrates that hard determinist intuitions, although a prominent position among scientists, have virtually no support in the general public.
